# Screening and identification of an anti-PD-1 nanobody with antitumor activity

**DOI:** 10.1042/BSR20221546

**Published:** 2023-01-19

**Authors:** Yanting Zhang, Shaoqi Yang, Dan Jiang, Yanning Li, Shuo Ma, Liyan Wang, Guangqi Li, Hongxia Wang, Aijun Zhang, Guangxian Xu

**Affiliations:** 1 General Hospital of Ningxia Medical University, Yinchuan, Ningxia 750004, China; 2 Department of Laboratory Medicine, School of Clinical Medicine, Ningxia Medical University, Yinchuan, Ningxia 750004, China; 3 Guangdong Provincial Key Laboratory of Medical Molecular Diagnostics, The First Dongguan Affiliated Hospital, Guangdong Medical University, Dongguan, Guangdong 523808, China

**Keywords:** check point immunotherapy, Nanobody, PD-1, phage display library

## Abstract

Blocking of programmed death protein 1 (PD-1) or its ligand PD-L1 with corresponding antibody to enhance T-cell response and mediate antitumor activity has been successfully applied in clinical practice. Several immune checkpoint inhibitors including monoclonal antibodies (mAbs) targeting PD-1 have been approved by the Food and Drug Administration (FDA) in cancer immunotherapy. However, the application of traditional antibodies has limited due to their drawbacks of large molecular weight (MW) and low tissue penetration. As the high specificity and strong tissue penetration of nanobodies (Nbs), efforts have been taken to develop Nbs for cancer therapy. Herein, we aim to screen a specific Nb against human PD-1 derived from a naïve camel Nb phage display library and further study its biological characteristic and antitumor activity. Finally, an anti-PD-1 Nb with high specificity and affinity was screened and generated, its cytotoxicity and antitumor effect was also confirmed *in vitro* and *in vivo*. All of these indicate that the anti-PD-1 Nb may provide an alternative and appealing therapeutic agent for cancer immunotherapy.

## Introduction

Immunotherapy has revolutionized the field of oncology [1]. Immune checkpoint inhibition has become one of the most important strategies in cancer treatment [2]. Programmed death protein 1 (PD-1) interacts with its ligand PD-L1 overexpressed on malignant tumor cells, leading to tumor immune evasion [3,4]. Blocking PD-1 or PD-L1 to enhance T-cell response and mediate antitumor activity has been successfully applied in clinical practice [5,6]. So far, several immune checkpoint inhibitors, including monoclonal antibodies (mAbs) targeting PD-1 or PD-L1, have been approved by the Food and Drug Administration (FDA) for cancer therapy [7]. While most patients benefit from the therapy, some do not respond to treatment and may develop severe side effects [8]. In addition, the application of traditional antibodies is affected by poor stability, large molecular weight (MW), low tissue penetration, and immune-related adverse events [9].

Nanobodies (Nbs), also known as the variable domain of the heavy chain of the heavy-chain-only antibody (VHH), with small size (∼15 kDa, 4 nm long, and 2.5 nm wide), high specificity, and strong tissue penetration, may provide an answer to these concerns. Nbs are composed of four framework regions (FR1–4) alternated with three complementarity determining regions (CDR1–3). The amino acid mutations in FR2 render the overall structure more hydrophilic, leading to higher solubility and possibly stability than conventional antibodies [10]. In addition, the extended CDR3 loop can recognize the hidden or buried epitopes, which cannot be recognized by traditional antibodies [11]. Due to the high similarity with the human VH sequence, Nbs have been associated with limited immunogenicity risks [12]. Studies have established the other hallmarks of Nbs, including high stability and chemical resistance [13,14]. Therefore, Nbs are suitable for fast selection from immune or naïve libraries and easy cloning based on the established display platform technology [15,16]. Additionally, Nbs could be easily produced in an appropriate expression system like *E. coli, S. cerevisiae*, or *P. pastoris*, with a favorable, cost-effective creation [17,18]. A recent study showed that an anti-PD-L1-specific Nb fused to human IgG1 Fc has remarkable antitumor activity *in vivo* and has been currently tested in clinical trials [19,20]. Another study demonstrated that a novel anti-PD-1 Nb expressed in yeast could block the PD-1/PD-L1 axis with potential immunotherapeutic effects [21]. These properties make Nb conducive to be a potential reagent in cancer immunotherapy.

In the present study, a specific Nb-targeting PD-1 was screened from a naïve camel Nb phage display library. The anti-PD-1 Nb was fused to human IgG1 Fc and expressed in a prokaryotic expression system. Its antitumor activity was then tested *in vitro* and *in vivo*.

## Materials and methods

### Generation of the VHH phage display library

The total RNA was extracted from the spleen of a healthy Bactrian camel and was well preserved in our lab using Trizol (Invitrogen, U.S.A.). The study has been approved by Medical Research Ethics Review Committee of General Hospital of Ningxia Medical University (KYLL-2021-195). The mRNAs were then transcribed to cDNA using reverse transcription PCR (ThermoScript RT-PCR kit, Thermo, U.S.A.). Then, the VHH fragments were amplified by two-step nested PCR using specific primers [22]. The primers of the first PCR were CALL001 and CALL002 (Supplementary Table 1). Amplicons spanning the VHH-CH2 exons with a size of 700 bp were generated during the first PCR with the common primers CALL001 and CALL002. Then, products (700 bp) were extracted from the 1.5% agarose gel as a template. The second PCR amplification was performed with VHH-Forward and VHH-Reverse primers (Supplementary Table 1). The final products (400 bp) were extracted from 2% agarose gel purification. As the second primer only coped with the VHH fragment, the fragments containing CH1 were removed. Next, a phage vector pCANTAB-5E and the amplified PCR products were digested with *NotI* and *SfiI* restriction enzymes (Takara, Japan) and then ligated with T4 DNA ligase enzyme (NEB, England) at a molar ratio of 1:3 at 16°C for 1 h at 4°C. The recombinant vector was electrotransformed into *E. coli* TG1 cells.

To produce phage, the library was cultured in 50-ml 2×YT medium (containing 0.1% glucose and 100 μg/ml ampicillin) at 37°C (OD = 0.5) and then incubated with 10^12^ M13O7 helper phage at 37°C for 30 min. To make VHH repertoire displayed on phage, library cells were collected and cultivated in 200 ml 2×YT medium supplemented with 100 μg/ml ampicillin and 50 μg/ml kanamycin at 30°C overnight. Samples were then collected and centrifuged at 9000 rpm for 10 min. The supernatant was mixed with 1/5 (v/v) PEG8000/NaCl for 8 h. Finally, the phage pellet was resuspended in PBS for later use. The size of the library was calculated by counting the number of colonies after gradient dilution. Finally, 20 colonies were randomly picked to estimate the VHH insertion rate of VHH genes by PCR amplification.

### Screening of PD-1 Nb by phage display

Based on the successfully constructed VHH library, biotinylated human PD-1 antigen (Acro biosystems, Newark) was used to select the specific coding anti-PD-1 VHH by phage display and soluble biopanning [23]. First, 500 μl phage and 5-μg-biotinylated hPD-1 antigen were mixed in a 1.5-ml centrifuge tube and rotated for 1 h at room temperature. Then, 50-μl streptavidin magnetic beads (Invitrogen, U.S.A.) were added and incubated for 30 min. The phage was eluted with 800 μl Tris-Hcl (pH 2.7) for 8 min and neutralized with 100 μl 0.2 M glycine (pH 9.1) after repeated washing. After that, the neutralized eluent was added to TG1 (OD600 = 0.5), incubated at 37°C for 30 min, and cultured on 2YT plates (supplemented with 2% glucose, 100 μg/ml ampicillin, and 50 μg/ml kanamycin) at 37°C overnight. The resulting phage was scraped for the next round of panning, in which the antigen protein was reduced to 3 μg. Ninety-six colonies were picked from the third- and fourth-round panning and cultured separately in 96 plates, then identified by Phage-ELISA. The supernatant of the colonies was collected and added to the plate wells, which were coated with human PD-1 protein (Acro biosystems, Newark) in advance for 1 h, followed by incubation with anti-M13/HRP (1:5000) antibody for another 1 h. The hydrogen peroxide and TMB were added to each plate well after repeated washing. The absorbance was read using Microplate Reader (Thermo, U.S.A.) at 450 nm. Finally, the positive colonies were sequenced and transformed into an amino acid sequence. CDR and FR were numbered according to IMGT [24]. The three-dimensional structure of the Nbs was predicted using a Swiss model. The MW and hydropathicity of the Nbs were evaluated using an online software of the ExPAsy-Protparam Tool.

### Construction and expression of recombinant PD-1 Nb

Two PD-1 Nb sequences (C11 and C12) were obtained after comparing the positive clones’ sequences using NCBI. Human IgG1 Fc gene fragment was linked to the PD-1 Nb sequence by PCR-based accurate synthesis. The anti-PD-1 Nb C12-Fc sequence was cloned into plasmid pCZN1 and then transformed into TOP 10 *E. coli* strain, while C11 Nb failed to ligate to pCZN1. The recombinant pCZN1-PD-1 Nb C12-Fc was then transformed into *E. coli* Arctic-Express and cultured at 37°C in LB medium containing ampicillin (50 μg/ml), after which it was induced with 0.5-mM isopropyl-β-d-thiogalactoside (IPTG). As the target protein (anti-PD-1 Nb-Fc) was mainly present in the precipitate (inclusion body), denaturing and refolding assay was performed. The protein was purified by Ni-NTA spin columns affinity chromatography (Novagen, Germany) and ultrafiltrated to remove the imidazole molecules and dialyzed into PBS solution. Ultimately, the purified anti-PD-1 Nb-Fc was checked by sodium dodecyl sulfate-polyacrylamide gel electrophoresis (SDS-PAGE).

### Affinity measurement

The affinity of obtained anti-PD-1 Nb-Fc was measured by surface plasmon resonance (SPR) using Biacore 8K (Healthcare Life Science, GE). First, the anti-PD-1 Nb-Fc (10 μg/ml) was immobilized on sensor chip CM5 (GE) to 300 RU with a flow speed of 10 μl/min. Then, the human PD-1 antigen (Acro biosystems) was injected into the experimental channel at a flow rate of 30 L/min with a concentration from 100 to 0.391 nM. The association and dissociation time was 120 and 300 s, respectively. The dissociation constant was calculated by Biacore Insight Evaluation Software (GE).

### PBMC isolation and CD4^+^ T cells enrichment

Blood samples were acquired from six volunteers (including three male and three female, their average age is 29.3 ± 2.53). The experiment was approved by the Ethics committee of Ningxia Medical University. Human peripheral blood mononuclear cells (PBMC) were isolated from EDTA anticoagulant blood by density gradient centrifugation with Human Lymphocyte Separation Medium (Solarbio, China). CD4^+^ T cells were enriched using magnetic bead separation (Miltenyi Biotec) according to the manufacturer’s instructions. The separated CD4^+^ T cells were then cultured in RPMI1640 medium (Biological Industries, U.S.A.) supplemented with 10% fetal bovine serum (FBS; BI) and Interleukin-2 (IL-2) 200 U/ml and activated by CD3/CD28 antibody (Peprotech, U.S.A.) 2 μg/ml in a humidified atmosphere containing 5%CO_2_/95% air at 37°C.

### Immunofluorescence staining

The role of anti-PD-1 Nb-Fc binding to PD-1 protein on human CD4^+^T cells was detected via immunofluorescence. Briefly, the activated CD4^+^ T cells were centrifuged at 1000 rpm for 15 min to immobilize on the polylysine-treated slides, and fixed with 4% paraformaldehyde, then treated with 0.1% Triton-X100. After being incubated with 5% bovine serum albumin (BSA), the slides were incubated with the anti-PD-1 Nb-Fc (50:1) and anti-PD-1 antibody (1000:1) overnight at 4°C. Finally, the slides were incubated with FITC-labeled goat antihuman secondary antibody (1:1000, Proteintech, China) for 1 h at room temperature and mounted with DAPI (Solarbio, China). Images were captured with a laser scanning confocal microscope (Olympus, Japan) and analyzed by ImageJ software. The experiment was repeated three times.

### ELISA

High-binding plates were coated with human PD-1 protein and mouse PD-1 protein overnight at 2 μg/ml, respectively. After being incubated with 5% BSA for 2 h, a serial diluted anti-PD-1 Nb-Fc was added to the coated plates at 37°C for 1 h. Then, samples were incubated with HRP-conjugated antihuman IgG at 37°C for 1 h after washing with PBST (PBS with 0.05% Tween-20). After additional washing, hydrogen peroxide and TMB were added to each well. The microplate reader measured absorbance at 450 nm.

The blocking effect of anti-PD-1 Nb-Fc on the interaction between PD-1 and PD-L1 was tested by competitive ELISA. High-binding plates were coated with PD-L1 (ACRO) overnight at 2 μg/ml in a coating buffer. After incubation with 5% BSA, biotinylated PD-1 protein (0.4 μg/ml) and a serial diluted anti-PD-1 Nb-Fc were added to the coated plates and incubated at 37°C for 1 h. Then, streptavidin-HRP (ACRO) and TMB were added after repeated washing. The microplate reader read absorbance at 450 nm.

### Primary T-cell activation assay

Freshly isolated PBMCs were treated by Staphylococcal enterotoxin B (SEB) serial dilutions (MCE, U.S.A.) and cultured at 10^5^ PBMCs/well in complete RPMI1640 for 72 h with anti-PD-1 Nb or unrelated control (anti-CD20 Nb-Fc produced in our lab, both with a concentration of 20 μg/ml). IL-2 levels in supernatants were measured using a human IL-2 ELISA Kit (BOSTER, China), according to the manufacturer’s instructions.

### Cell lines

RKO, SW620, HT29, DLD1, SW480, HCT116, and HEK-293T (an embryonic kidney cell line) were purchased from ATCC. RKO, SW620, HT29, DLD1, SW480, and HCT116 were maintained in RPMI1640 supplemented with 10% FBS (BI) and 1% penicillin-streptomycin (Solarbio, China), while the HEK-293T cells were cultured in DMEM (BI, U.S.A.). All cells were cultured in a humidified atmosphere containing 5%CO_2_/95% air at 37°C.

### Western blot

Total proteins of activated human T cells and 293T cells were extracted using RIPA lysis (KenGen Biotech, China). The proteins were separated on 10% SDS-PAGE gel and transferred to polyvinylidene difluoride membranes (Millipore, U.S.A.). After blocking in 5% nonfat dry milk in TBS-Tween20 for 2 h, the membranes were incubated with anti-PD-1 Nb-Fc (1:500) overnight at 4°C. Then, the membranes were washed in TBS-Tween20 and incubated with HRP-conjugated antihuman IgG secondary antibody (1:5000) at room temperature for 1 h. Protein bands were visualized with ECL (Thermo Fisher, U.S.A.) and detected on a chemiluminescence detection system (Bio-Rad, U.S.A.).

### Flow cytometry

All the colorectal cancer cell lines were cultured and harvested to detect the expression of PD-L1 by flow cytometry. Briefly, tumor cells (5 × 10^5^) were harvested and stained with 20 μl PE-conjugated antihuman PD-L1 antibody (BD, U.S.A.) at room temperature and protected from light for 30 min. Afterward, the prepared samples were measured and analyzed by Cyto FLEX flow cytometer (Beckman coulter, U.S.A.). RKO cells that highly express PD-L1 were selected in the following research. RKO cells with luciferase activity were well established and screened using puromycin for 14 days.

### Cytotoxicity to tumor cells *in vitro*

Cytotoxicity *in vitro* was evaluated by luciferase report assay. Human PBMCs were incubated overnight with 200 U/ml IL-2, and used as effector cells. Mitomycin C-treated RKO cells were used as target cells. The effector cells to target cells ratio (E:T) was set as 40:1, 20:1, 10:1, and 5:1, respectively. The PBMCs and RKO cells were cocultured for 24, 48, and 72 h at 37°C with RPMI1640 and 10% FBS in 96-well plates. Serial dilutions of anti-PD-1 Nb-Fc, unrelated control Nb-Fc, or anti-PD-1 mAb were then added. The fluorescence was detected with Single-Luciferase Report Assay Kit (TransDetect, China) and measured by Microporous Plate Detector. The percentage of cytotoxicity was calculated by the following formula:

$$1-\left(\frac{experimental\;values}{\text{max}\;values}\right)$$

### Xenograft mice model

All animal experiments took place at Laboratory Animal Center of Ningxia Medical University. The work has been approved by the Institutional Animal Ethical and Welfare Committee of Ningxia medical University. The ethics approval number is IACUC-NYLAC-2021-010. A 5- to 8-week-old female NOD/SCID mice (*n*=35, five mice per group) were purchased from Vital River Laboratory Animal Technology Co., Ltd., Beijing, China (license number: SCXK Beijing 2016-0006). All mice were raised in a specific pathogen-free environment and received sterile drinking water and food. All animal studies were done in compliance with the regulations and guidelines of Ningxia Medical University institutional animal care and conducted according to the AAALAC and the IACUC guidelines. The mice were under isoflurane anesthesia in noninvasive bioluminescent imaging experiment. At the end of the experiment, the mice were sacrificed by cervical dislocation.

Human PBMCs were cultured in RPMI1640 supplemented with 10% FBS and IL-2 for 5 days. RKO cells were then mixed with PBMCs at a 2:1 ratio. Cell mixtures were subcutaneously injected into the right flank of NOD/SCID mice. The mice were then randomized into the following groups: untreated group (subcutaneous injection with RKO cells only), PBMC group (PBMCs and RKO cells), PBMC + control Nb-Fc group (PBMCs and RKO cells, and treated with unrelated Nb-Fc), PBMC + anti-PD-1 Nb-Fc-treated groups, and PBMC + mAb group. Anti-PD-1 Nb-Fc, unrelated control Nb-Fc, or anti-PD-1 mAb were administered intraperitoneally on day 1, 4, 7, and 10 at the indicated doses. Tumor growth was measured with a standard caliper, and the tumor volume was calculated using the formula: (width^2^ × length × 0.52).

As the RKO cells were engineered to express luciferase, noninvasive bioluminescent imaging (BLI) was also used to evaluate the tumor burden. The mice were intraperitoneally injected with luciferin (Promega, U.S.A., 150 μg/kg) and then imaged under isoflurane anesthesia using Lumina III Living Image System (Perkin Elmer) every 9 days postimplantation. Living Image Software (Perkin Elmer) captured and analyzed the images. On day 28, the mice were sacrificed by cervical dislocation after anesthesia.

### Statistical analysis

GraphPad 8.0 Software was used for statistical analysis. Data are shown as the mean ± standard error and analyzed by one-way ANOVA. The comparison of tumor volume was analyzed by a Mann–Whitney rank-sum test. *P*<0.05 was considered statistically significant.

## Results

### Generation of the VHH phage display library

**Figure 1:**
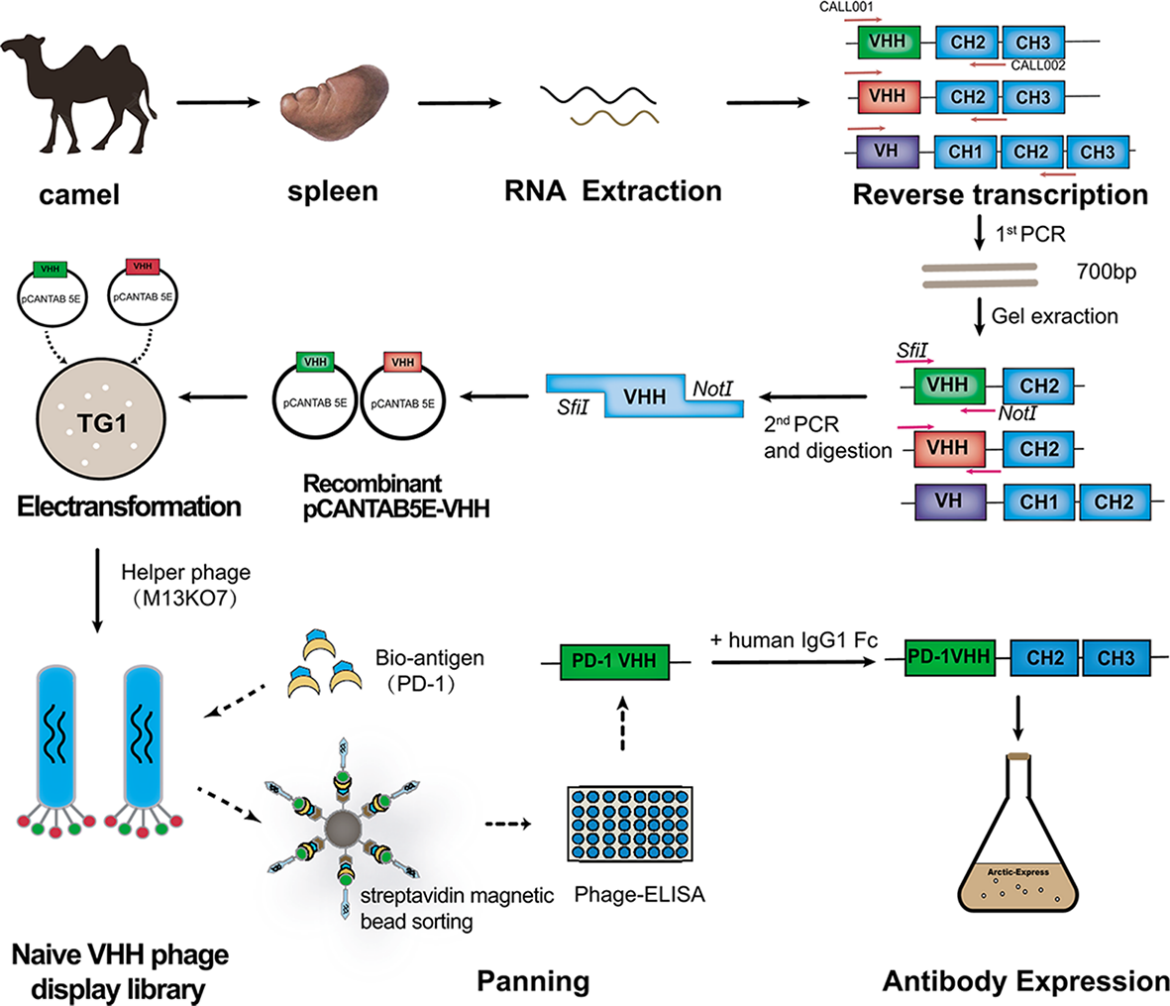
Generation of VHH phage display library, screening, and development of anti-PD-1 Nb

The strategy to construct anti-PD-1 Nb-Fc is shown in Figure 1. Briefly, total RNA was extracted from the spleen of a healthy Bactrian camel and reverse transcribed to cDNA. Using cDNA as template, the VHH gene fragment was amplified by two-step nested PCR and then cloned into a pCANTAB5E phage vector (Supplementary Figure S1A,B). The recombinant pCANTAB5E-VHH was transformed into *E. coli* TG1 to construct a naïve VHH phage display library. After rescuing by helper phage M13KO7, the size of the library reached 5.1 × 10^14^ CFU/ml (Supplementary Figure S1D), which could satisfy the following studies. The 20 individual colonies were randomly selected for PCR analysis, and the result showed a VHH genes insertion rate of 100% (Supplementary Figure S1C).

### Selection of anti-PD-1 Nb

**Figure 2:**
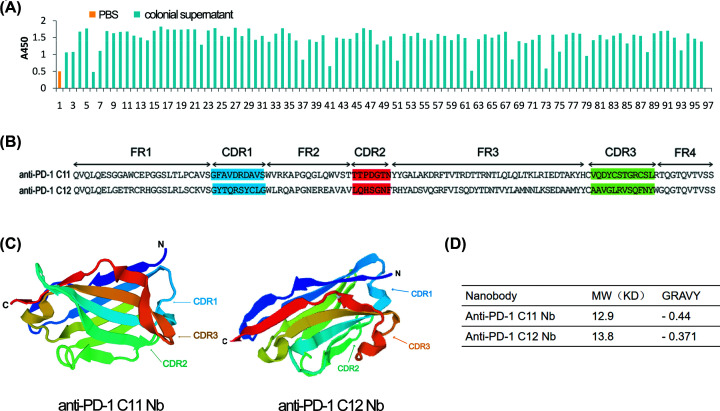
Screening of anti-PD-1 Nb (**A**) The positive colonies from the third and fourth panning round were further selected by Phage-ELISA. The supernatant of the colonies was added to the plates coated with the PD-1 antigen, and the anti-M13/HRP was added as the secondary antibody. The positive candidates with absorbance greater than two times that of the negative control (PBS) were selected. (**B**) The amino acid sequences of anti-PD-1 Nbs C11 and C12. Amino acids position of CDR and FR are numbered according to IMGT. CDR1, CDR2, and CDR3 are shown in blue, red, and green, respectively. (**C**) The 3D structure of the Nbs was predicted using a Swiss model. (**D**) The MW and the grand average of hydropathicity (GRAVY) of the Nbs were also evaluated using an online software of ExPAsy-Protparam Tool.

The anti-PD-1 Nbs were screened from the rescued naïve Nb library by biotinylated PD-1 antigen and streptavidin magnetic beads (Figure 1). After four rounds of panning, monoclonal positive colonies were further selected by Phage-ELISA (Figure 2A). The positive candidates were identified with absorbance more than two times that of the negative control. Twelve clones with the highest absorbance value were subjected to sequencing. After comparing the gene sequences of the positive clones in NCBI, two anti-PD-1 Nbs (C11 and C12) were identified. The amino acid sequences were translated from the nucleotide sequence by the online tool ExPASy-Translate Tool. FR and CDR were divided according to the IMGT scientific chart (Figure 2B). The predicted 3D structure and feature of the Nbs showed that the Nb structure consists of two β sheets, and the MW of Nbs is 15 kd with favorable hydropathicity (Figure 2C,D).

### Construction and expression of the anti-PD-1 Nb

**Figure 3:**
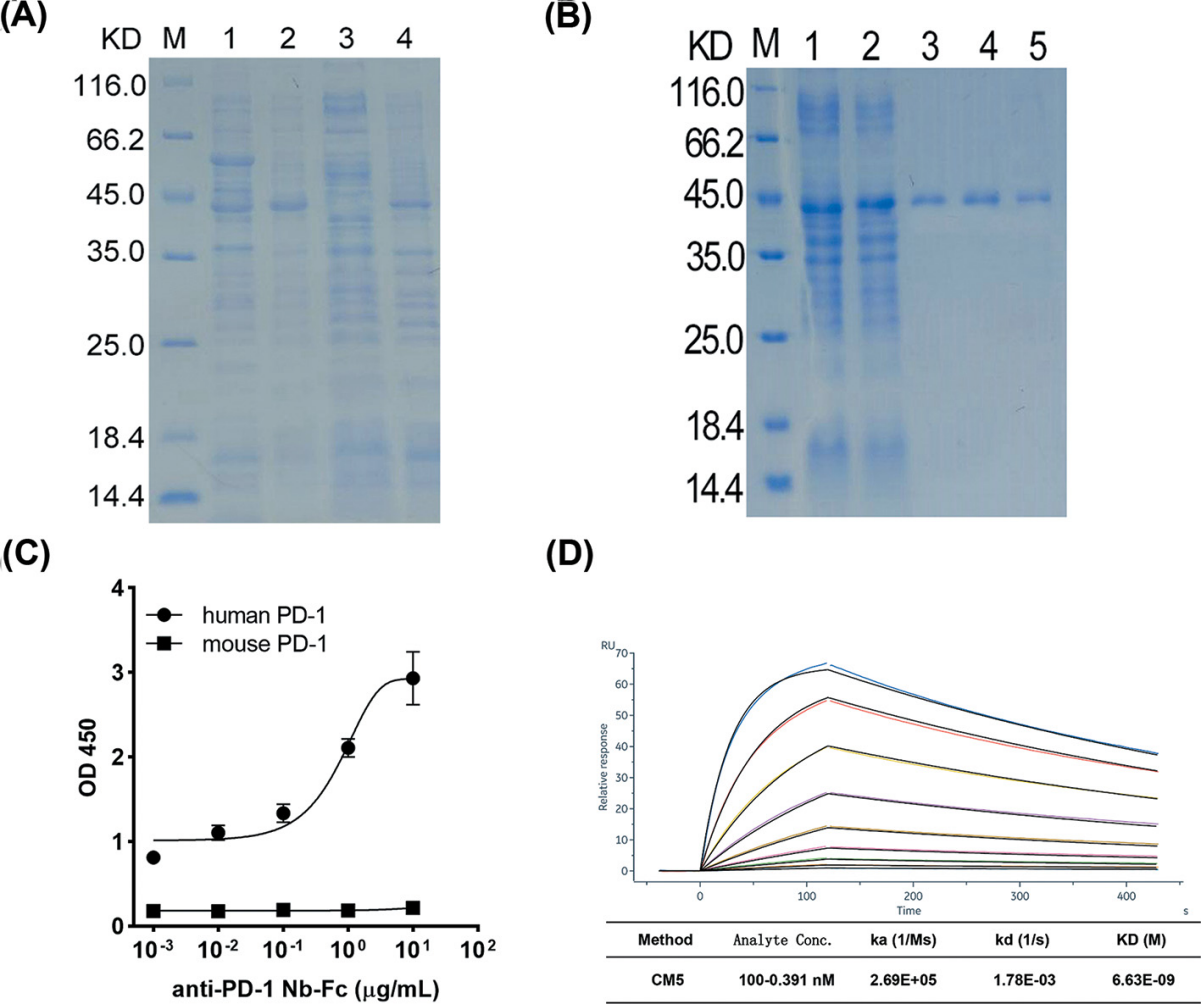
Expression of anti-PD-1 Nb-Fc and its affinity to PD-1 protein (**A**) SDS-PAGE analysis of anti-PD-1Nb-Fc expression. M: protein marker; Lane 1: before IPTG induction; Lane 2: after IPTG induction; Lane 3: supernatant after sonication; Lane 4: precipitate after sonication. The recombinant anti-PD-1Nb-Fc was detected in the precipitate of the culture. It was dissolved after denaturing and refolding. (**B**) SDS-PAGE analysis of the purified anti-PD-1 Nb-Fc. M: protein maker; Lane 1: precipitate after sonication; Lane 2: effluent during purification; Lanes 3–5: eluate after purification. The MW of the obtained anti-PD-1 Nb was about 40.1 KD. (**C**) The initial binding capacity of anti-PD-1 Nb-Fc to human PD-1 and mouse PD-1 protein was detected by ELISA. (**D**) The affinity of anti-PD-1 Nb-Fc was measured by SPR. The equilibrium dissociation constant (KD) is 6.63 × 10^−9^ M.

As the anti-PD-1 Nb sequence came from a naïve camel Nb library with the MW of 15 kd, which would be rapidly eliminated from circulation, we fused the anti-PD-1 Nb to human IgG1 Fc fragment by PCR-based accurate synthesis to prolong the effective half-life. The pCZN1-PD-1 C12 Nb-Fc vector was successfully recombined and transformed into TOP 10 *E. coli*, while the recombination of anti-PD-1 C11 Nb-Fc with pCZN1 failed. The pCZN1-PD-1 C12 Nb-Fc vector was transformed into *E. coli* Arctic-Express to express the target protein, anti-PD-1 Nb-Fc. After induction with IPTG, the anti-PD-1 Nb-Fc was detected in the precipitate of culture (Figure 3A). The anti-PD-1 Nb-Fc was then dissolved in PBS after denaturing and refolding. SDS-PAGE analysis showed a band of 40 kDa after purification by Ni-NTA spin columns affinity chromatography (Figure 3B). These findings suggested that the recombinant anti-PD-1Nb-Fc was successfully expressed in a prokaryotic system. We finally obtained highly purified recombinant Nb for the following research.

### Anti-PD-1 Nb-Fc has high affinity to PD-1 protein

The binding capacity of obtained anti-PD-1 Nb-Fc was tested by ELISA (Figure 3C). Anti-PD-1 Nb-Fc could bind to human PD-1 antigen at a low concentration of 1 ng/ml while was unable to combine with mouse PD-1 antigen, which indicated that there was no cross interaction with mouse PD-1. The affinity was further tested by SPR. The result showed that the equilibrium dissociation constant (KD) of anti-PD-1 Nb-Fc is 6.63 nM, suggesting a high binding ability to PD-1 (Figure 3D).

### Anti-PD-1 Nb-Fc has high specificity and activity *in vitro*

**Figure 4:**
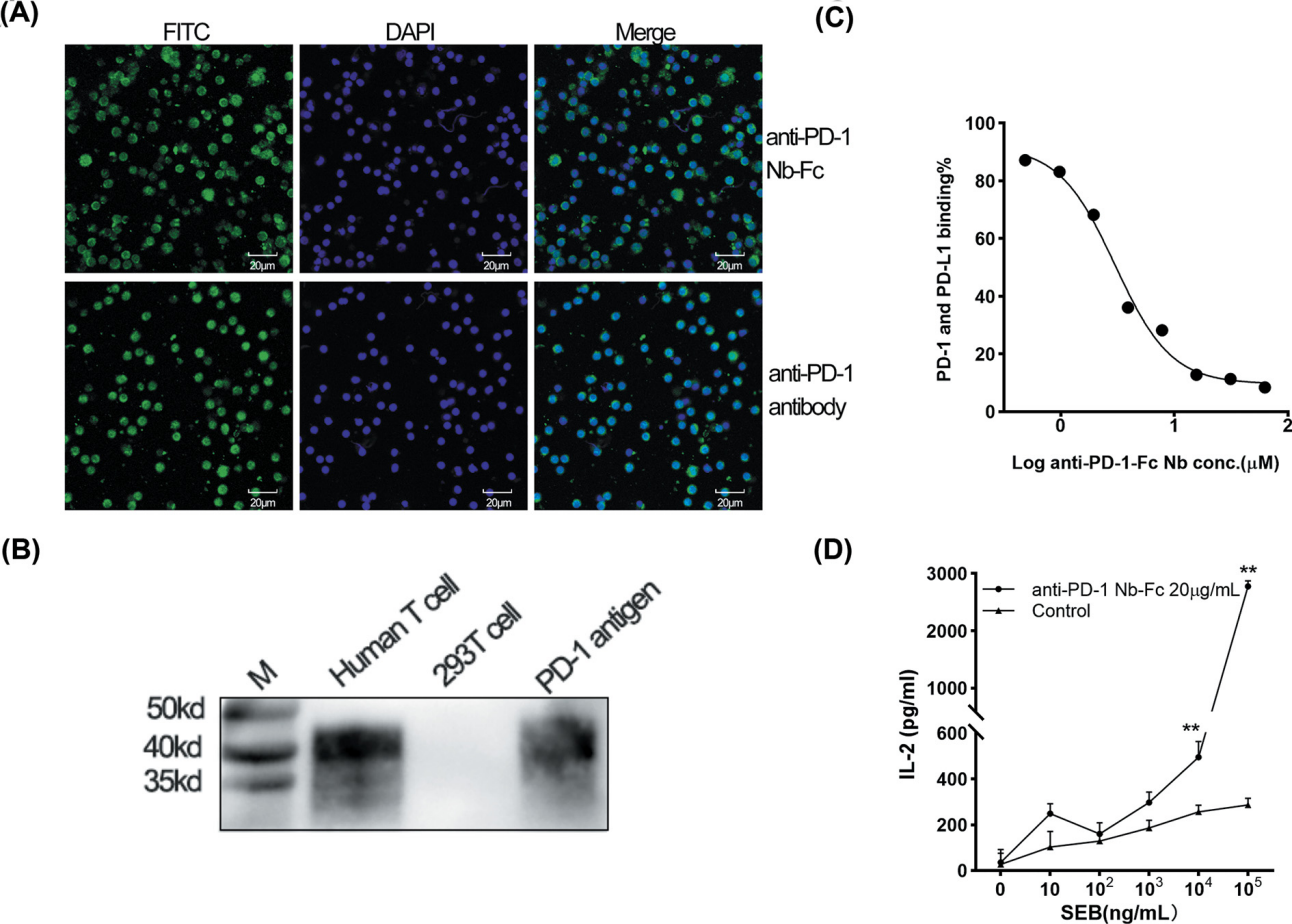
Specificity and activity of the anti-PD-1 Nb-Fc *in vitro* (**A**) Immunofluorescence was used to analyze the cell-binding capacity of anti-PD-1 Nb-Fc. Anti-PD-1 Nb-Fc and anti-PD-1 antibody were used as the primary antibody; cells were then stained by the FITC-labeled secondary antibody (green), while the cell nucleus was stained with DAPI (blue). Original magnification, ×400. (**B**) The specificity of anti-PD-1 Nb was analyzed by western blot. Anti-PD-1 Nb was used as the primary antibody, HRP-conjugated antihuman IgG as a secondary antibody. The anti-PD-1 Nb-Fc recognized PD-1 antigen (recombinant human PD-1 protein) and PD-1 protein expressed on activated human T cells. There was no band on 293T cell protein and nonactivated human T cells. (**C**) The blocking effect of anti-PD-1 Nb-Fc on the interaction between PD-1 and PD-L1 was detected by ELISA. IC_50_ is 3.015 mM. (**D**) Primary T-cell activation assay. Anti-PD-1 Nb-Fc could enhance IL-2 secretion over control (unrelated anti-CD20-Fc Nb produced in our lab) in response to SEB using PBMCs *in vitro*. The addition of anti-PD-1 Nb-Fc increased IL-2 secretion about tenfolds,** *P*≤0.01.

The cell-binding capacity of anti-PD-1 Nb-Fc was revealed by immunofluorescence staining. In the presence of anti-PD-1 Nb-Fc, the green fluorescence was detected on the surface of the activated CD4+ T cells, which implied that anti-PD-1 Nb-Fc could bind to PD-1 protein expressed on activated human CD4+ T cells (Figure 4A). The specificity of anti-PD-1 Nb-Fc was further analyzed by Western blot. The anti-PD-1 Nb-Fc could recognize PD-1 protein expressed in activated human T cells (Figure 4B).

We further tested its inhibitory effect on the interaction between PD-1 and PD-L1 through competitive ELISA (Figure 4C). As a result, anti-PD-1 Nb-Fc was able to inhibit the PD-1/PD-L1 interaction effectively.

To determine the ability to overcome PD-1 checkpoint suppression in T cells, the anti-PD-1 Nb-Fc was evaluated in a primary T-cell activation assay. The addition of anti-PD-1 Nb-Fc increased IL-2 secretion by about tenfolds compared with its control group, suggesting that the anti-PD-1 Nb-Fc could enhance the function of human T cells by increasing IL-2 secretion in response to SEB (Figure 4D). These findings suggest that anti-PD-1 Nb-Fc could bind to activated human T cells and block PD-1/PD-L1 interaction to further active T cells.

### Anti-PD-1 Nb-Fc could enhance the antitumor effect *in vitro*

**Figure 5:**
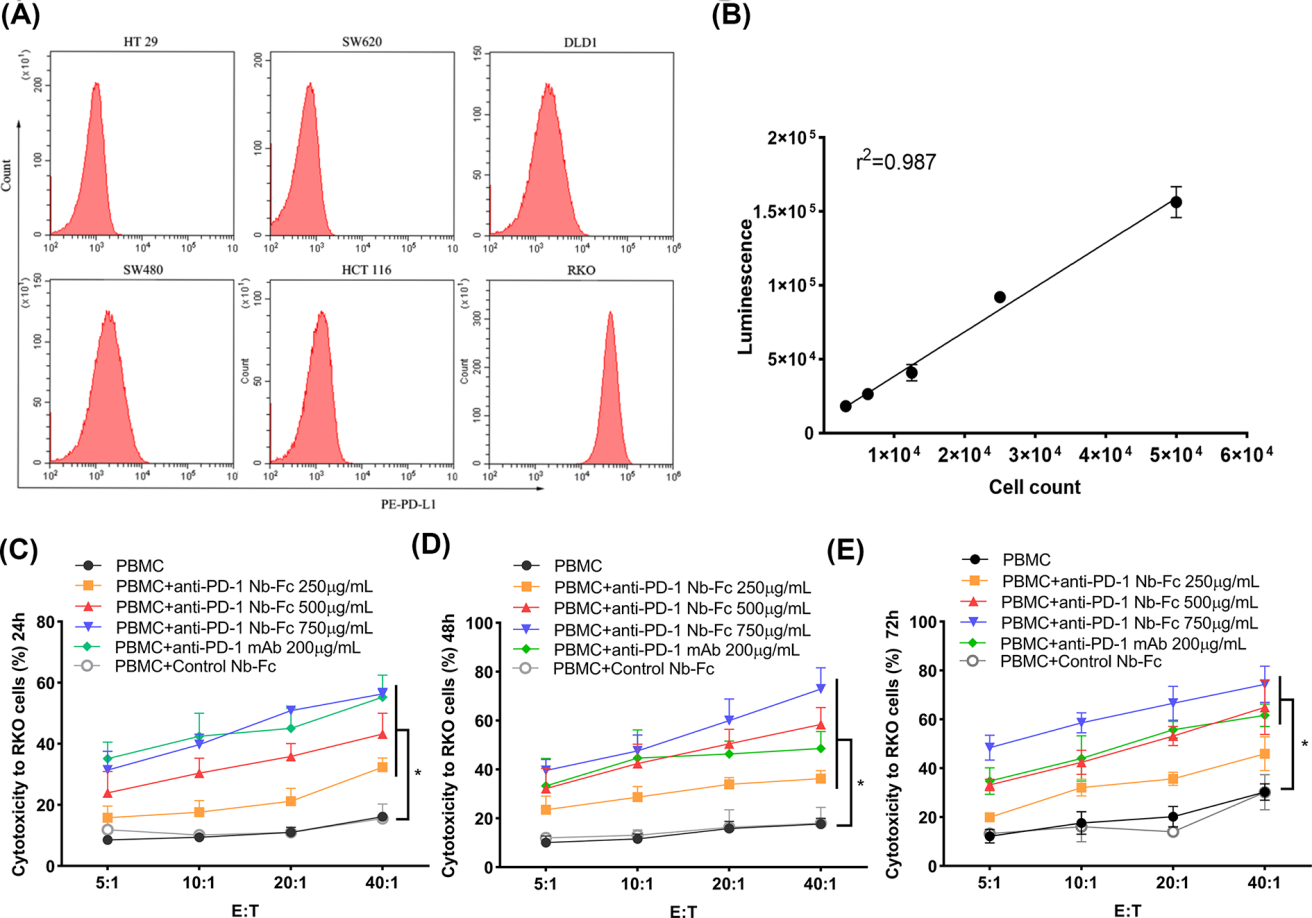
The anti-PD-1 Nb-Fc enhanced cytotoxicity of PBMC to tumor cells *in vitro* (**A**) The expression of PD-L1 in colorectal cancer cell lines (including HT29, SW620, DLD1, SW480, HCT116, and RKO cells) was analyzed by flow cytometry. RKO cells were finally selected for further research considering they have the highest PD-L1 expression vs other cell lines. (**B**) RKO cells stably express luciferase. The wild RKO cells were transfected with a lentiviral vector encoding the firefly luciferase gene; stable expression was obtained using 14 days of puromycin selection. (**C–E**) Cytotoxicity of anti-PD-1 Nb-Fc on tumor cells. Different concentrations of anti-PD-1 Nb-Fc, anti-PD-1 mAb, or control Nb-Fc (unrelated anti-CD20-Fc Nb produced in our lab) were added to the coculture system of PBMCs and RKO cells with different E/T ratio and interaction time, *P≤0.05.

The cytotoxicity of anti-PD-1 Nb-Fc was investigated using RKO cells, which highly express PD-L1 (Figure 5A). In order to exactly calculate the cytotoxicity, RKO cells, engineered to express firefly luciferase (Figure 5B), were cocultured with human PBMCs in different E/T ratio and treated with different concentrations of anti-PD-1 Nb-Fc, anti-PD-1 mAb, or control Nb-Fc. The cytotoxicity was significantly increased when treated with anti-PD-1 Nb-Fc (250, 500, and 750 μg/ml) and mAb compared with the PBMC group at 24 and 48 h, while there was no difference between the control group and PBMC group (Figure 5C,D). Furthermore, the cytotoxicity of anti-PD-1 Nb-Fc (500 and 750 μg/ml) and mAb was significantly higher compared with that of the PBMC group at 72 h (Figure 5E). The cytotoxicity of anti-PD-1 Nb-Fc with 750 μg/ml was the highest in all time points, although there was no statistical significance compared with the mAb group. Meanwhile, the cytotoxicity of every group increased when the E/T ratio was increased. Hence, the anti-PD-1 Nb-Fc could enhance the antitumor effect *in vitro*.

### Anti-PD-1 Nb-Fc could inhibit tumor growth *in vivo*

We further evaluated the efficiency of anti-PD-1 Nb-Fc *in vivo*. As the therapeutic mechanism of the anti-PD-1 antibody was to block the PD-1/PD-L1 pathway to enhance the antitumor T-cell effector activity, we established the xenograft mice model of human colorectal cancer [25]. RKO cells mixed with human PBMCs at a ratio of 2:1 were subcutaneously inoculated into the right flank of NOD/SCID mice. The untreated group received only RKO cells, while the PBMC group was inoculated with a mixture of RKO cells and PBMCs. Then, anti-PD-1 Nb-Fc, control Nb-Fc, or mAb were administered intraperitoneal on day 1, 4, 7, and 10 postcell injection.

**Figure 6:**
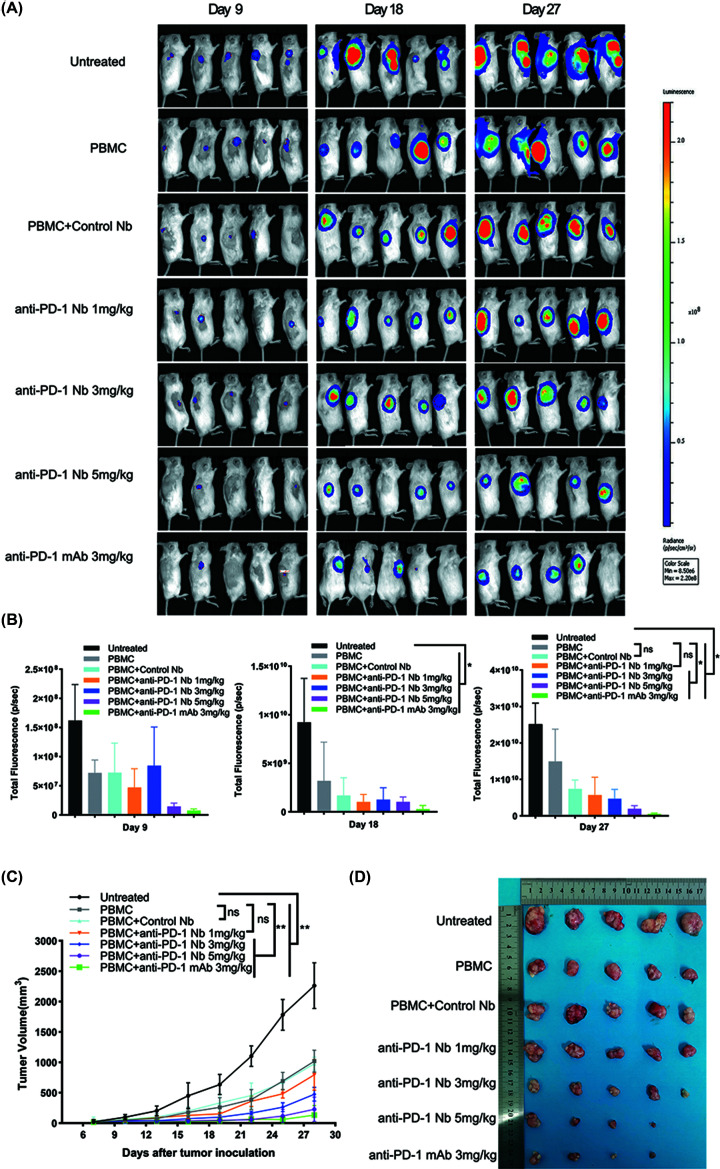
The antitumor activity of anti-PD-1 Nb-Fc in xenograft mice model of human cancer (**A**) BLI was used to assess disease progression on day 9, 18, and 27 after implantation. (**B**) The total radiance of the tumor was analyzed by Living Image Software, *P≤0.05. (**C**) The tumor volumes (mm^3^) in NOD/SCID mice, **P≤0.001. (**D**) Physical map of tumor tissues.

To exactly observe the disease progression, mice were monitored with BLI on day 9, 18, and 27 (Figure 6A). There was no difference in tumor luminescence on day 9. However, the luminescence in the PBMC group, anti-PD-1 Nb-Fc (1, 3, 5 mg/kg) group, and mAb group were significantly lower compared with the untreated group at day 18 (*P*<0.05). On day 27, the luminescence in anti-PD-1 Nb-Fc (3 and 5 mg/kg) group and mAb group was lower than that in the PBMC group (*P*<0.05), while there was no difference between PBMC and the control Nb-Fc group (*P*>0.05) (Figure 6B). This implied that anti-PD-1 Nb-Fc could reduce the tumor burden of the colorectal cancer xenograft mice. Moreover, the tumor volume in treated groups (including the PBMC group, anti-PD-1 Nb-Fc group, control Nb-Fc group, and mAb group) was much smaller compared with the untreated group (*P*<0.05). Also, the tumor burden in mice treated with anti-PD-1 Nb-Fc (3 and 5 mg/kg) and mAb was remarkably smaller compared with the PBMC group (*P*<0.05). Tumor growth inhibition of anti-PD-1 Nb-Fc with 5 mg/kg was most obvious than in other concentrations (*P*<0.05). There was no difference between the PBMC group and the control Nb group (*P*>0.05) (Figure 6C).

All the mice were sacrificed after 28 days; tumors were collected and photographed. Similarly, the tumor size in treated groups was much smaller than in the untreated group, and in anti-PD-1 Nb-Fc and mAb groups were smaller than in the PBMC group and the control Nb-Fc group (Figure 6D). Accordingly, anti-PD-1 Nb-Fc significantly inhibits the tumor growth of RKO xenografts (*P*<0.05).

## Discussion

PD-1 is a key surface receptor on T lymphocytes that modulates the immune response. PD-1 inhibitors unblock the immune suppression of antitumor T cells, which results in T-cell proliferation and infiltration to enhance antitumor response [26,27]. Antibody blockade of PD-1 has been confirmed to enhance antitumor immune therapy in various cancers [28–30]. However, large MW and low tissue penetration limit its application.

Nbs are becoming promising diagnostic and therapeutic proteins in oncology due to their unique functional and structural properties. In the present study, we screened and developed a specific Nb-targeting PD-1 from a naïve camel Nb phage display library and demonstrated its antitumor activity *in vitro* and *in vivo*.

Several routes have been used to screen specific Nbs, including generating a VHH library [31,32]. The key point in screening a specific Nb with high affinity is the capacity of a phage display library. A naïve phage display library could avoid a tedious animal immune process using less time and cost than the immune library [33]. In the present study, the naïve camel-derived phage display library was structured with fresh camel spleen, rendering the capacity much richer than that with peripheral blood; its capacity reached 5.1 × 10^14^ CFU/ml, which could satisfy the requirement of selection. By a series of panning in the naïve phage display library, an anti-PD-1 Nb was successfully selected and sequenced.

The small size of VHH enables easier tissue penetration. But, it also results in a short half-life. The general strategy to prolong the half-life is fusing VHH with long-circulating serum proteins like albumin or Fc fragment [34,35]. In the present study, we fused the screened anti-PD-1 Nb to a human IgG1 Fc fragment to prolong its effective half-life *in vivo* and maintain its penetration stability to some extent.

Nbs could be easily produced in an appropriate expression system like *E. coli, S. cerevisiae*, or *P. pastoris*, with a favorable, cost-effective creation. The recombinant anti-PD-1Nb-Fc was successfully expressed in a prokaryotic system.

The recombinant anti-PD-1 Nb-Fc could specifically bind to human PD-1, but not to mouse PD-1, with high affinity. It also has an inhibitory effect on the interaction between PD-1 and PD-L1, which lead to further T-cells activation. Based on the favorable feature, the antitumor effect of anti-PD-1 Nb-Fc was evaluated *in* *vitro* and *in* *vivo*. It effectively enhances T-cell functions and cytotoxicity against RKO cells *in* *vitro*. Although the recombinant anti-PD-1 Nb-Fc could not avoid tumor formation in Xenograft mice model, it could significantly delay tumor growth, indicating that the anti-PD-1 Nb-Fc could inhibit tumor growth *in* *vivo*.

We successfully screened and developed an anti-PD-1 Nb-Fc with high specificity, affinity and antitumor effect. These findings indicate that the anti-PD-1 Nb-Fc may be used as an alternative and appealing therapeutic agent for cancer immunotherapy.

However, some issues need to be understood before it is used as clinical therapeutic agents in cancer treatment. First, despite the anti-PD-1 Nb-Fc exerting well antitumor effect *in vitro* and *in vivo*, it needs a high dose to function. According to previous studies, a viable method to increase the potency of Nbs is to assemble them into multimers or bispecific constructs [36,37]. Based on the favorable characteristic of the anti-PD-1 Nb-Fc, designing a multivalent or multispecific Nb to enhance its potency is worth to be further investigated. Second, since its generation involves use of bacteriophages, in future research for human clinical therapeutic reagent, safety concerns need to be well evaluated. Besides, we have not studied the serum half-life of anti-PD-1 Nb-Fc as well as its concentration in tumor. We will further explore its pharmacokinetics in future.

## Supplementary Material

Supplementary Figure S1 and Table 1

## Data Availability

The data from the present study are available from the Corresponding Author upon request.

## Abbreviations

BLI: bioluminescent imaging
BSA: bovine serum albumin
CDR: complementarity determining region
DAPI: 4',6-diamidino-2-phenylindole
FBS: fetal bovine serum
FDA: Food and Drug Administration
FR: framework region
IL-2: Interleukin-2
IPTG: isopropyl-β-d-thiogalactoside
mAb: monoclonal antibody
MW: molecular weight
Nb: nanobody
NOD/SCID: nonobese diabetic severe combined immunodeficiency
PBMC: peripheral blood mononuclear cell
PD-1: programmed death protein 1
PD-L1: programmed death ligand 1
SDS-PAGE: sodium dodecyl sulfate-polyacrylamide gel electrophoresis
SEB: Staphylococcal enterotoxin B
SPR: surface plasmon resonance
VHH: variable domain of the heavy chain of the heavy-chain-only antibody
