# Supplementary material for: Screening and identification of an anti-PD-1 nanobody with antitumor activity
Source: Biosci Rep. 2023 Jan 19;43(1):BSR20221546. doi: 10.1042/BSR20221546 (PMC9867944; doi:10.1042/BSR20221546)
Supplement: Supplementary Figure S1 and Table 1 [file BSR-2022-1546_supp.pdf]

Name	Sequence(5′ to 3′)
CALL001	GTCCTGGCTGCTCTTCTACAAAG
CALL002	GGTACGTGCTGT TGAACTGTTCC
VHH-Forward	TCGCGGCCCAGCCGGCCCAGGTCCAACTGCAGGAGTCTGGGG
VHH-Reverse	ATAAGAATGCGGCCGCTGAGGAGACGGTGAC CTGGGTCCCC

Supplementary Table 1 Sequences and applications of primers used in this study.

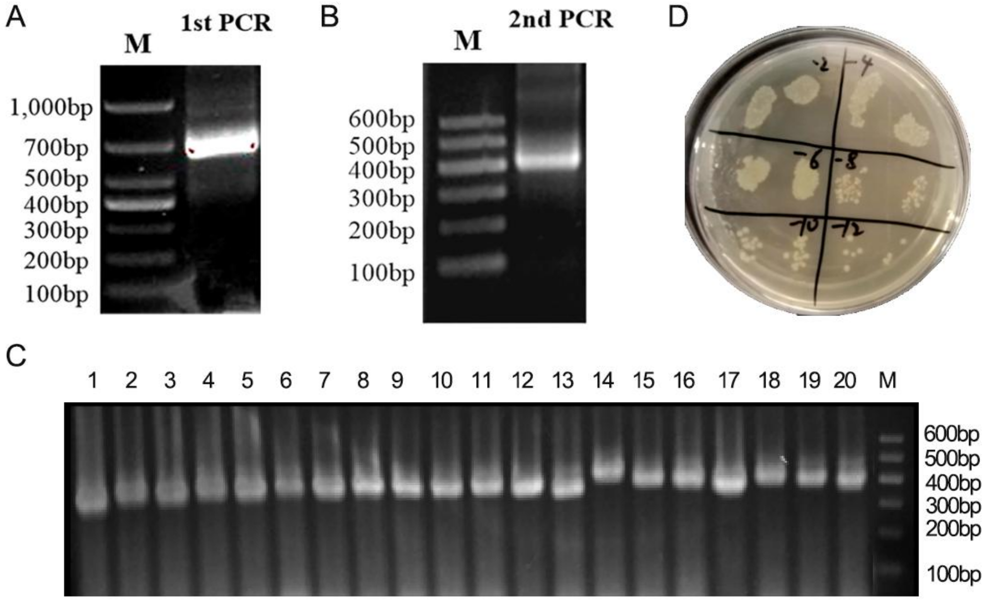

Supplementary Fig.1 Generation of the naïve phage display library.

(A-B) VHH genes were obtained by Nested PCR. **(C)** 20 colonies were randomly picked to estimate the correct insertion rate of VHH genes by PCR amplification. **(D)** The library size was measured by counting colonies number after serial dilution.
